# Pathogenic and Diagnostic Potential of BLCA-1 and BLCA-4 Nuclear Proteins in Urothelial Cell Carcinoma of Human Bladder

**DOI:** 10.1155/2012/397412

**Published:** 2012-07-02

**Authors:** Matteo Santoni, Francesco Catanzariti, Daniele Minardi, Luciano Burattini, Massimo Nabissi, Giovanni Muzzonigro, Stefano Cascinu, Giorgio Santoni

**Affiliations:** ^1^Department of Medical Oncology, Polytechnic University of the Marche Region, 60126 Ancona, Italy; ^2^Division of Urology, Department of Clinical and Specialist Sciences, Polytechnic University of the Marche Region, 60126 Ancona, Italy; ^3^Section of Experimental Medicine, School of Pharmacy, University of Camerino, Via Madonna delle Carceri, 62032 Camerino, Italy

## Abstract

Transitional cell carcinoma (TCC) of the bladder is one of the most common malignancies of genitourinary tract. Patients with bladder cancer need a life-long surveillance, directly due to the relatively high recurrence rate of this tumor. The use of cystoscopy represents the gold standard for the followup of previously treated patients. Nevertheless, several factors, including cost and invasiveness, render cystoscopy not ideal for routine controls. Advances in the identification of specific alterations in the nuclear structure of bladder cancer cells have opened novel diagnostic landscapes. The members of nuclear matrix protein family BLCA-1 and BLCA-4, are currently under evaluation as bladder cancer urinary markers. They are involved in tumour cell proliferation, survival, and angiogenesis. In this paper, we illustrate the role of BLCA-1 and BLCA-4 in bladder carcinogenesis and their potential exploitation as biomarkers in this cancer.

## 1. Background

Transitional cell carcinoma (TCC) represents more than 90% of bladder cancers [1], ranking among genitourinary malignancies only behind prostate cancer for frequency and estimated mortality. At initial diagnosis, more than 70% of bladder tumors are confined to the mucosa or lamina propria. Transurethral resection of nonmuscle invasive tumors can be accompanied by intrabladder therapy, depending on tumor depth and grade. However, more then 70% of patients can present tumor recurrences after treatment, with up to 30% of patients progressing to higher tumor stage and grade [2]. 

In this view, close and accurate disease surveillance is essential for monitoring tumour recurrence and progression to invasive disease. The current standard diagnostic iter includes urine cytology, imaging, and flexible cystoscopy. Cytology represents the cornerstone of urine-based bladder cancer diagnosis. It involves microscopic examination of precancerous and cancerous cells present in the urine by a pathologist. Although its high specificity (96%), the sensitivity is lower (44%) [3], particularly for low-grade tumors [4]. 

Quanticyt is a karyometric of bladder washing for the quantitative grading of urine cytology [5]. Based on the DNA content levels and nuclear morphometry, bladder cancer can be classified into low, intermediate, and high risk of recurrence [6]. Emerging data from the main studies involving the use of Quanticyt showed that this test has a sensitivity of 56.4% (range 42.1–69%) and a specificity of 72.1% (range 67.9–76%) [7, 8].

The use of cystoscopy has been successful in monitoring bladder cancer recurrence [9]. On the other hand, cystoscopy is not ideal for the life-long followup of patients with bladder cancer, considering its cost and invasiveness; moreover, the difficulty in identifying asymptomatic patients has prompted the search for more reliable noninvasive markers for the early detection of bladder cancer.

Noninvasive urine-based markers represent a novel diagnostic approach. BCLA-1 and BCLA-4 are included in this list that also comprises nuclear matrix protein 22 (NMP22) and bladder tumour antigen (BTA). 

In 1996, Getzenberg et al. identified six bladder-specific nuclear structure proteins (BLCA-1 to 6), expressed exclusively by bladder cancer cells [10]. These nuclear matrix proteins (NMPs) are involved in several functions, including DNA replication, RNA synthesis, and nuclear morphology. This review describes the functional role played by BLCA-1 and BLCA-4 in bladder carcinogenesis, illustrating the currently available data concerning their potential diagnostic employ.

## 2. Functional Role of BLCA-1 and BLCA-4 in Bladder Carcinogenesis

Changes in nuclear structure can affect gene expression, thus playing an important role in the carcinogenesis process [11]. In 1977, BerezneyandCoffey first described the nuclear matrix structure [12]. It is composed by protein components derived from three structural regions: a lamina with nuclear pores, the residual nucleoli, and an internal matrix framework connected to a residual nuclear layer containing pore complexes. Nuclear matrix represents an active environment where DNA replication [13, 14] and RNA synthesis take place [15, 16]. NMPs recognize and bind to specific DNA sequences called scaffold/matrix attachment regions (S/MAR), partitioning DNA into functional loop domains. S/MARs are involved in chromosomal replication, transcription, recombination, and condensation. They interact with topoisomerase II, identified by Berrios et al. in 1985 as a major polypeptide component of the Drosophila nuclear matrix-pore complex-lamina fraction [17]. The S/MAR interacting elements also include lamins A and C [18], Poly(ADP-ribose)polymerase 1 and 2 (PARP-1, PARP-2) [19], and CCCTC-binding factor (CTCF) [20] that binds to the regulatory regions of *c-myc* gene [21]. Moreover, certain S/MARs require adjacent transcription factors to become active [22]. Therefore, nuclear morphology is deeply influenced by NMPs. Based on these findings, NMPs have been investigated as potential cancer markers. Moreover, the discovery that NMPs are released into urine and serum has suggested their exploitation for cancer diagnosis.

NMP-22 (NuMA) is a 239 kDa nuclear matrix protein located in the mitotic spindle [23]. It is involved in microtubule assembly and in the partitioning of genome into newly formed G1 nuclei during cellular replication [24]. NMP22 is released from cells undergoing apoptosis [25]. NMP22 levels are higher in bladder cancer cells as compared to the normal counterpart [26]. NMP22 test has been the first nuclear matrix-based test approved by the U.S. Food and Drug Administration (FDA) for the followup of bladder cancer patients and for the screening of patients with suspected symptoms or with a family history of bladder cancer. Sensitivity of NMP22 test varies from 68.5% to 88.5% and specificity varies from 65.2% to 91.3% depending on the chosen cut-off [27–32]. Positive and negative predictive values of NMP 22 test vary from 34% to 76% and from 77.9% to 98%, respectively [28–31].

Evaluation of NMP22 has been also used for the diagnosis of colorectal carcinoma [33] and as a rejection marker in kidney transplantation [34]. 

BLCA-1 was originally identified from bladder tumor tissue [10]. While BLCA-4 is detected also in normal adjacent tissue, BLCA-1 is only expressed by tumor cells, suggesting their different role in bladder carcinogenesis.

BLCA-1 gene is similar to TI-227H, a cancer metastasis-associated gene discovered in 1996 by Ishiguro et al. [35] and isolated from the B16-F10 mouse melanoma subline [36]. The homology with TI-227H gene suggested a potential role of BLCA-1 to discriminate metastatic versus localized disease. However, no relation has been found so far between BLCA-1 levels and advanced bladder cancer.

In 2005, Myers-Irvin et al. [37] examined BLCA-1 expression in bladder tissue and urine via Western blot and ELISA, respectively. They observed that BLCA-1 protein levels were significantly higher in patients with bladder cancer than in normal individuals but did not correlate with tumor grade. They registered 80% sensitivity and 87% specificity, demonstrating the potential employment of a BLCA-1-based assay in diagnosis and surveillance of patients with bladder cancer.

The gene that encodes BLCA-4 was sequenced by Getzenberg et al. in 2004. They observed common regions (a nuclear localization sequence and helix-loop-helix region) with the *ELK-3* gene, a member of the ETS transcription family [38]. These factors bind to DNA, specifically interacting with C/A GGA A/T sequences. ETS transcription factors are involved in apoptosis, carcinogenesis, VEGF expression, and angiogenesis, and local and metastatic diffusion [39]. 

Elk-3 is phosphorylated and activated by the Ras-extracellular signal-regulated kinase (Erk) pathway. Together with Elk-1 and Sap-1, Elk-3 forms the ternary complex transcription factor subfamily (TCF), which regulates the early response of quiescent cells to growth factor stimulation [40]. BLCA-4 interacts with several known transcription factors, such as AP-1, AP-2, NFATc, NF-E1, and NF-E2 [38]. In 2005, Myers-Irvin et al. analyzed the BLCA-4 functions by transfecting the *BLCA-4* gene into four different human bladder carcinoma cell lines. BLCA-4-transfected bladder cancer cells exhibited a growth advantage as compared to untransfected cells. Additionally, microarray analysis revealed an upregulation of genes related to cell growth, including the cyclins, as well as of interleukin-8 (IL-8), thrombomodulin (TM), and interleukin-1alpha (IL-1*α*) in overexpressing BLCA-4 cells. Analysis of the *IL-1*α**, *IL-8*, and *TM* gene promoters evidenced multiple ETS sites in their sequence, suggesting that BLCA-4 binds directly to each gene to cause overexpression [41].

IL-8 has been shown to play an important role in multiple cellular processes. In 1997, Thalmann et al. observed that urinary IL-8 was a prognostic factor of bladder cancer recurrence and progression after Bacillus Calmette-Guerin therapy [42]. In 2002, Lang et al. described the signaling pathway regulating IL-8-supported bladder carcinoma cell migration. In 2008, Chikazawa et al. observed in an orthotopic model of murine bladder cancer that tumorigenicity, angiogenesis, and metastasis formation were significantly higher for tumor cells expressing higher IL-8 levels [43]. This increased tumor growth and metastasis formation could be attributed to the IL-8-mediated up-regulation of the metalloproteinases MMP-2 and MMP-9 expression and activity [44]. In 2009, Tseng-Rogenski and Liebert suggested an additional role for IL-8 as a growth and essential survival factor for normal human urothelial cells. They observed that exogenous human recombinant IL-8 promoted normal urothelial cell growth through the activation of Akt pathway [45]. 

The important role of IL-8 in bladder cancer is also suggested by the results obtained by Milan et al. [44]. They tested the ability of a fully human anti-IL-8 antibody, ABX-IL8, to affect TCC growth *in vitro* and in an *in vivo* mouse model. They observed a significant decrease in tumor growth accompanied by downmodulation of the nuclear factor-kappaB (NF-*κ*B) expression and transcriptional activity, and consequently in significant inhibition of MMP-2 and MMP-9 expression. 

IL-8-251 T > A polymorphism also seems to be a relevant susceptibility factor for bladder carcinoma development and to influence bladder cancer patients' outcome after BCG immunotherapy [46]. 

Finally, recent studies suggest a potential role of IL-8 in malignant transformation of urothelial cells [47, 48]. 

IL-1 is an inflammatory cytokine, present in two different isoforms, IL-1*α* and IL-1*β*, with similar biological functions. The role of IL-1 in bladder cancer tumorigenesis and angiogenesis is still unclear. IL-1 can promote bladder cancer cell adhesion and increase the expression of matrix degrading enzymes, thus favoring tumor invasion. However, IL-1*α* has also been reported to reduce tumor angiogenesis and participate in the regulation of immune responses. These data were confirmed by Seddighzadeh et al., who observed a correlation between low IL-1*α* messenger RNA levels and decreased overall survival (OS) in patients withurinarybladdercarcinoma [49]. 

TM is a cell-surface-expressed glycoprotein, predominantly synthesized by vascular endothelial cells [50]. TM is a necessary anticoagulant factor in the protein C pathway and is also involved in inflammation, fibrinolysis, apoptosis, cell adhesion, and cellular proliferation [51–53]. BLCA-4 over-expression results in increased TM expression, which sustains the microcirculation [54] needed for tumor cell survival.

In 2000, Konety et al. [55] detected 53 out of 55 samples of histology proven bladder carcinoma cases through the high level of BLCA4 in urine, without registering any false positivity in 51 normal controls. In addition, in 2005 Van Le et al. [56] developed a sandwich immunoassay based on two BLCA-4 antibodies directed against distinct epitopes. BLCA-4 was measured in precipitated urine samples from patients divided into four different groups. Group A consisted of patients with bladder cancer, groups B and C included patients with various benign urologic conditions, group D was composed by patients with prostate cancer, and group E with healthy individuals. The mean BLCA-4 level for the patients in Group A was significantly higher than the mean of the other groups. Thus, the indirect BLCA-4 immunoassay showed a specificity of 95% and a sensitivity of 89%.

## 3. Conclusion

Bladder cancer is the fourth most common cancer in men and the ninth most common in women. Despite recent advances, the molecular mechanisms underlying bladder carcinogenesis are still not fully elucidated so far. The screening of high-risk population has become critical to diminish the mortality. Current methods used for a proper diagnosis of bladder cancer mainly rely on cystoscopy, which can be associated with biopsy or resection. The importance of detecting bladder cancer at the early stage is clearly demonstrated by the 94% of 5-year survival rate registered in patients with localized disease. Cytology has represented for decades the noninvasive standard for the detection of urinary bladder cancer cells. However, cytology lacks sensitivity, particularly for low-grade tumors. In the last years, the list of identified urinary markers in course of evaluation has been rapidly enlarged. Preliminary data on emerging markers have shown higher sensitivity than routine urinary cytology, especially when a panel of markers were used. The urinary markers and tests under investigation include BTA, NMP22, BLCA-1, BLCA-4, hyaluronic acid, hyaluronidase, cytokeratin-8, cytokeratin-18, cytokeratin-19, telomerase, Immunocyt, Quanticyt, FDP, FISH, and CYFRA-21-1.

 At present, the role of BLCA-1 in bladder carcinogenesis has not been clarified. The homology with the TI-227H metastasis-associated gene suggests BLCA-1 a potential candidate to discriminate metastatic versus localized disease. The use of BLCA-1 as urinary marker of bladder cancer has provided promising results, being observed a sensitivity and a specificity of 80% and 87%, respectively. Moreover, further studies are required to investigate the correlation of BLCA-1 expression with tumor grade and clinical stage, considering the small number of low-grade tumors in the study by Myers-Irvin et al. 

As regards to BLCA-4, results show that its expression does not only provide bladder cancer cells with growth advantage but can also cause malignant cell transformation. BLCA-4 activity is mediated by IL-1, IL-8, and thrombomodulin, which can act by maintaining blood flow for tumor cell survival, enhancing tumor cell proliferation and invasion, and increasing tumor angiogenesis.

Among urinary markers for bladder cancer, BLCA-4 has registered the highest sensitivity and specificity. Its potential is further strengthened by the absence of high BLCA-4 levels in patients with various benign urologic disorders, such as urinary tract infection, catheterization, or cystitis. Nevertheless, novel methods not requiring urine precipitation analysis are necessary to include BLCA-4 assay into clinical practice.

In conclusion, preliminary data suggest that BLCA-1 and BLCA-4 show a high potential as bladder cancer urinary markers. Their employ may aid to identify individuals in the early stages of bladder cancer, thus affecting the therapeutic approach and prognosis of these patients. Further studies are required to evaluate the role of the BLCA-1 and BLCA-4 assays in the detection of this disease.
